# Complications of a Unilateral Nondisplaced Cervical Facet Fracture in a Patient With Previously Noninstrumented Anterior Cervical Fusion

**DOI:** 10.5435/JAAOSGlobal-D-21-00067

**Published:** 2021-07-07

**Authors:** Mohammed A. Munim, Innocent U. Njoku, Christina W. Cheng

**Affiliations:** From the Case Western Reserve University School of Medicine (Munim and Dr. Cheng), and the Department of Orthopaedic Surgery (Dr. Njoku and Dr. Cheng), University Hospitals Cleveland Medical Center, Cleveland, OH.

## Abstract

Fused motion segments have been documented to alter the biomechanics of the cervical spine and compromise its stability. Current literature describes a growing association between the presence of prior noninstrumented fused cervical segments and the predisposition to acute, traumatic instability at adjacent levels. We present the case of a stable cervical spine fracture pattern in a patient with a history of multilevel noninstrumented anterior cervical spine fusion—initially presenting as a small, nondisplaced unilateral facet fracture that ultimately progressed to overt displacement with kyphosis resulting in acute cervical pain and instability. The patient underwent urgent open reduction and instrumented posterior fixation. We discuss the challenges associated with a timely diagnosis and offer insight into the surgical management of this rare yet potentially catastrophic complication.

Anterior cervical diskectomy and fusion (ACDF) is the benchmark treatment for degenerative cervical spine disease, first reported in the 1950s by Robinson and Smith^1^ and Cloward.^2^ Through its evolution since, this procedure has demonstrated widespread success for both one-level and multilevel decompression and fusion, providing >90% likelihood of substantial relief of radicular and myelopathic symptoms.^3,4^ Many studies have even compared the exact technique of fusion—ACDF with and without instrumentation—and justified the use of one or the other for certain indications. Instrumented fusions, using fixation devices such as anterior cervical plating, have become increasingly popular as recent studies report the benefit of added stability at the bone-graft interface, which consequently has shown to increase multilevel fusion rates and decrease revision surgery rates.^5^ However, the use of instrumented ACDF for single-level fusions remains controversial, as other studies have highlighted that internal fixation may interfere with graft consolidation in the disk space.^6^ It seems that for these cases, noninstrumented ACDF, using diskectomy or bone grafting alone, offers greater benefit at a lower cost. The comparison between the two techniques is further highlighted when deliberating the postoperative outcomes of cervical spine fusions. There seems to be an association between the presence of fused noninstrumented cervical segments and the predisposition to acute traumatic instability. Mac Millan and Stauffer found delays in the diagnoses of acute traumatic fractures through preexisting cervical fusions resulting from aberrations in atypical fracture patterns.^7^ Consequently, the presence of fusions significantly affected the treatment decisions in this unique cohort. As the number of fused segments increases, a progressively longer lever arm is necessitated, thereby increasing the biomechanical demands at neighboring levels in the cervical spine. Consequently, this produces less technically successful fusions.^6,7^

As such, patients with preexisting noninstrumented multilevel fusions may exhibit unsatisfactory levels of bone and soft-tissue healing that make them high risk for accelerated cervical spine instability. Several authors have described the development of new degenerative changes that tend to occur immediately adjacent to a prior fused segment—typically within one or two levels above or below. Many of these chronic changes were demonstrated to act as catalysts for acute cervical spine fractures when involved in a traumatic setting, requiring emergent surgical fixation.^7,8^ Moreover, one recent study has even described major traumatic acute displacement and fracture-dislocations directly through a fused segment, in contrast with previous studies reporting fractures in adjacent areas.^9^ We report on a second such case of a patient who had an initial nondisplaced fracture, which subsequently displaced through a previously robust noninstrumented multilevel anterior cervical fusion. To our knowledge, this is the first report in medical literature of a case with such a rare injury pattern and timeline. The presence of a prior noninstrumented fusion should raise suspicion for instability after cervical spine trauma. The objective of this report is to describe a rare atypical fracture pattern to demonstrate factors affecting misdiagnosis and provide a discussion on of the diagnostic workup to identify such cases and prevent delays in management.

## Case Report

**Figure 1:**
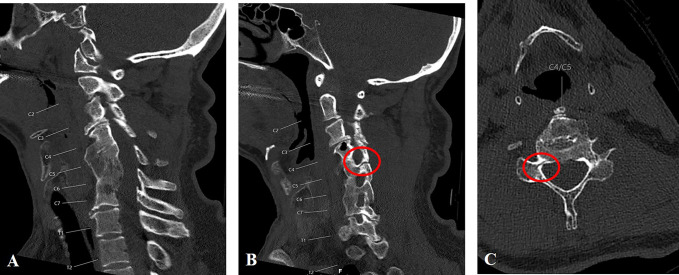
Initial sagittal CT scan demonstrating (**A**) previous anterior cervical fusion mass between C4 to C7 and (**B**) a subtle nondisplaced facet fracture line at the right C4-C5 level, extending into the C5 right pedicle as indicated by the red circle. **C**, Axial cut at C4-C5 with subtle fracture line through the right C5 pedicle as indicated by the red circle.

**Figure 2:**
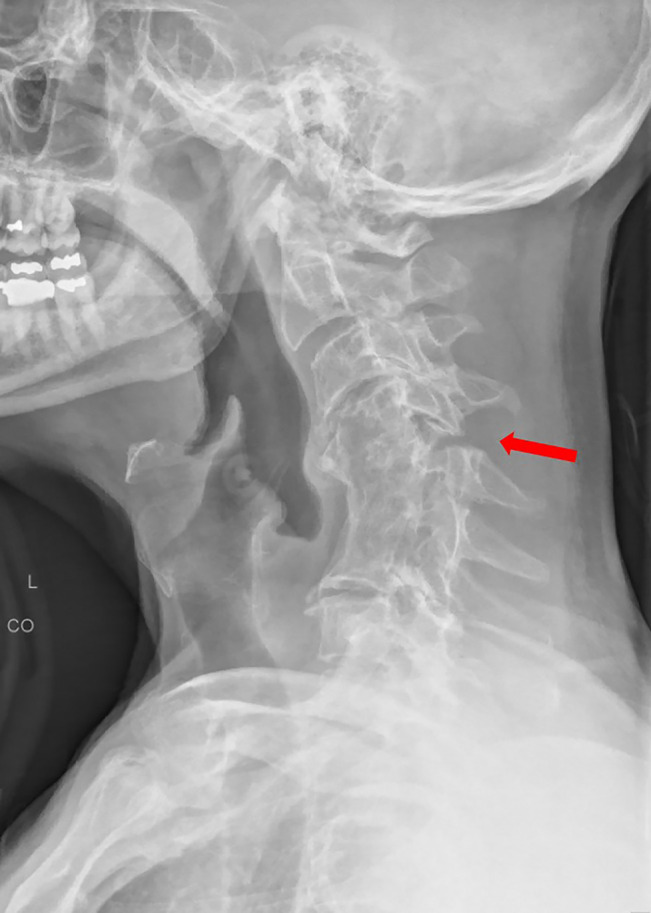
Preoperative standing upright lateral cervical spine radiograph demonstrating cervical kyphosis and flexion-distraction at the C4-C5 facet, suggestive of an unstable spine as indicated by the red arrow.

A 62-year-old man with a history significant for concussions and early Alzheimer disease initially presented to the emergency department for neck pain after sustaining an unwitnessed ground-level fall at home. Given the patient’s history of cognitive decline, the exact mechanism of his low-energy traumatic event/fall is unclear. His surgical history revealed that he had underwent a prior, successful noninstrumented anterior cervical fusion from C4 to C7 to treat cervical radiculopathy more than 20 years ago. Initial CT of the cervical spine in the emergency department showed a lucency likely suggestive of a small fracture through the right C4-C5 facet extending into the right C5 pedicle, with no displacement (Figure 1). The stability of the fracture was likely underappreciated because CT only provides a supine view of the fracture without the presence of gravity to challenge the stability of the fracture. The CT did not demonstrate widening between the spinous process or facets or increase kyphosis of the cervical spine that would be suggestive of cervical instability. In the emergency department, the patient was placed in a Philadelphia collar and discharged home. At his follow-up clinic appointment 10 days later, the patient reported persistently severe neck pain in conjunction with new radicular pain along his left arm. He also reported slight paresthesia in the left upper extremity that did not follow any particular dermatomal distribution. Examination demonstrated ataxic gait. He had five out of five strength in both upper and lower extremities with intact sensation throughout and absent Hoffman sign and had normal Babinski reflex. His American Spinal Injury Association Impairment scale was grade E. Upright cervical spine radiographs, both AP and lateral views, were subsequently obtained in clinic. The lateral view demonstrated obvious distraction across the C4-C5 facet with focal kyphosis (Figure 2), suggestive of an unstable fracture at risk for further displacement with the potential of spinal cord injury and paralysis. The decision was made to proceed with urgent posterior cervical instrumented fusion and stabilization from C2 to T2.

**Figure 3:**
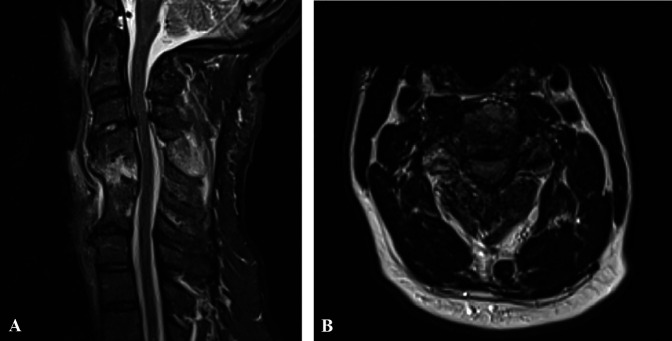
**A**, Preoperative sagittal Short Tau Inversion Recovery-weighted MRI revealed local bone marrow edema at the C4-C5 anterior fusion and posterior ligamentous structures. **B**, Axial section through C3-4 demonstrating severe stenosis of the spinal canal.

A preoperative MRI of the cervical spine was completed because the patient reported left cervical radiculopathy with mild symptoms of gait imbalance. MRI demonstrated bone marrow edema in the C5 vertebral body and right pedicle. This edema corresponded to a subtle fracture line originating at the site of the C4-C5 fusion, extending through the right C5 pedicle. In addition, imaging demonstrated severe stenosis at C3-C4 immediately superior to the fusion (Figure 3). Of note, spinal cord stenosis was observed at the superior adjacent level of the fractured segment, which likely is associated with the increased kyphosis, injury to the posterior ligamentous complex, and transosseous disruption at the fractured level. We elected to treat the fracture with a posterior instrumented cervical fusion from C2-T2 with laminectomy and decompression of the stenosis at C3-4. A posterior approach would allow for stabilization of the fracture with posterior instrumentation and decompression and avoid complications associated with revision surgery through an anterior approach. In addition, given that the fracture involves the posterior elements, additional fusion anteriorly would not correct this, and therefore, posterior fusion would be the most stable fixation.

Once patient was in agreement to surgery and written consent was obtained, he was taken to surgery. Under general anesthesia, a Mayfield head holder was placed on the patient and he was placed in a prone position, and the cervical spine was positioned in a neutral alignment. A midline incision was placed, and a subperiosteal dissection was performed down to the cervical spine. Cervical spine levels were confirmed with fluoroscopy before instrumentation. Once exposed, fracture was seen through the right C4-5 facet; however, the posterior ligamentous complex was intact. We then proceeded with instrumentation with bilateral pars screws at C2. Lateral mass screws were placed bilaterally from C3 to C6, with the exception of the right C5 lateral mass at the site of the fracture. Pedicle screws were placed bilaterally at T1 and T2. All screw positioning was carefully performed under fluoroscopic guidance and confirmed with AP and lateral images. The patient then underwent a C3-C4 laminectomy without complications.

Two titanium rods contoured to the cervical lordosis were screwed in sequentially. We then compressed the right C4-C6 screws to reduce the fracture. All connections between the screws and rods were tightened and torqued appropriately; intraoperative fluoroscopy assessed reduction and verified acceptable screw position and rod alignment. Morcellized bone graft consisting of autologous lamina-harvested bone as well as Demineralized Bone Matrix allograft fiber and chronOS tricalcium phosphate was applied to the decorticated C2-T2 posterior lateral bone elements. Electrodiagnostic potentials were stable and consistent throughout the entire case with no intraoperative complications noted.

Postoperatively, the patient was placed in a rigid cervical collar. No immediate complications were observed, and postoperative upright radiographs and CT of the cervical spine confirmed acceptable reduction and implant placement. The patient had a slightly prolonged hospital stay due to poor pain tolerance, but he was ultimately discharged home 3 days postoperatively.

**Figure 4:**
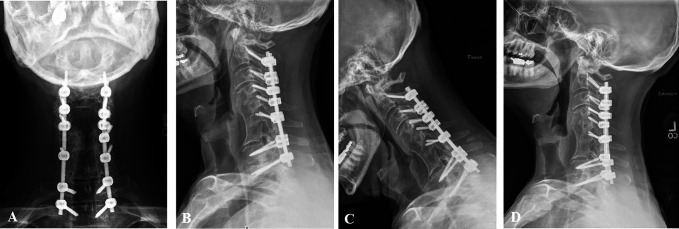
**A**, AP, (**B**) lateral, (**C**) flexion, and (**D**) extension radiographs obtained 3 months after surgery showing C2-T2 posterior spinal fusion with instrumentation, fracture reduction, and restored anatomic alignment. The patient did not report neck pain, arm pain, or neurologic signs of spinal cord compression.

At 1-month follow-up, the patient reported no axial neck pain. He reported mild right shoulder pain with overhead activity and forward flexion. At 3 months after surgery, the patient reported feeling much better and was no longer required pain medications. His right shoulder pain had completely resolved, and his left arm pain had markedly improved. Standing upright AP, lateral, and flexion-extension views of the cervical spine demonstrated stable alignment of the cervical spine with solid maintenance of the C4-C5 reduction, C2-T1 posterior fusion, and instrumentation at 3 months (Figure 4).

## Discussion

Reported evidence exists—both clinically and in biomechanical studies—which demonstrates that fused segments in the cervical spine have significant effects on the remaining free motion segments. However, the literature is limited in understanding the effects of atypical fractures through a noninstrumented multilevel cervical fusion; such injuries are scarce as they are either underreported or underdiagnosed. We report a case of the gradual displacement of an initially nondisplaced unilateral cervical facet fracture in a patient with previously noninstrumented anterior cervical spine fusion. Open reduction with instrumented posterior cervical fusion corrected the cervical deformity and stabilized the fracture to avoid further progression and risk of acute spinal cord injury. Previous literature has established how fused segments in the cervical spine predispose it to the effects of future acute trauma. It is postulated that the biomechanically compromised nature of fused constructs weakens the cervical spine, yielding it to be incapable of handling the stress, pressure, and motion it is naturally accustomed to.^9^ Another consideration demanding attention is poor healing of the existing fusion that may have impaired bone quality in the cervical spine. This deterioration is comparable to what is seen in patients with inflammatory conditions such as ankylosing spondylitis (AS), diffuse idiopathic skeletal hyperostosis (DISH), and Klippel-Feil syndrome. However, for these, the fracture patterns are diversified depending on the inherent pathologic process: patients with AS tend to develop transdiscal fractures due to tardy ossification of the nucleus pulposus, whereas DISH fractures commonly involve the vertebral body.^10-12^ Patients with Klippel-Feil syndrome typically present with areas of instability immediately adjacent—within two levels—of the fusion.^7^ Fracture patterns caused from minor or even no trauma typically involve both anterior and posterior complexes and can be significant enough to produce instability associated with neurologic deficit.

Irrespective of the cause, management of these fractures parallels the challenges seen in those with previous noninstrumented ACDF. Various authors have reported that the use of external mobilization alone is frequently ineffective, leading to poor immobilization rates and loss of reduction.^8^ When surgical management is pursued, rigid fixation may be difficult secondary to osteoporosis and deformed vertebrae. To this end, many authors have advocated the use of long posterior fusions with multiple-point internal fixations encompassing numerous levels (>5) above and below with subsequent use of a cervical collar.^13,14^ As was seen in our patient, cervical pedicle and lateral masses may be too osteoporotic to function as acceptable anchor points for fixation. In such circumstances, we found that successful stabilization requires lengthening the fusion both proximally into the upper cervical spine and distally into the thoracic spine until adequate fixation purchase is secured.

In patients with prior noninstrumented ACDF, the apparent complication of acute traumatic cervical instability is somewhat uncommon, with a reported incidence of 5.4%.^15^ A handful of reports in the literature exist. Mac Millan and Stauffer reviewed 15 cases of traumatic cervical instability, three of which occurred in patients with noninstrumented ACDF. Instability manifested in the form of facet dislocations immediately adjacent to the level of the fusion, which were all eventually treated conservatively with external immobilization.^7^ The authors postulated that posterior ligamentous structures have less biomechanical strength in tension than their anterior counterparts, which may explain the higher susceptibility to traumatic injury in anterior fusions. Raizman et al^15^ reported a single case of a 55-year-old woman with C5-C6 noninstrumented ACDF who developed a C4-C5 facet dislocation with posterior disk herniation after a motor vehicle accident; treatment involved decompression and open reduction with an instrumented C4-C5 fusion. Yoshihara et al^16^ described a patient with a C3-C7 ACDF who had an acute C7/T1 vertebral body fracture and facet dislocation after a fall. Most recently, Yokoyama et al^3^ presented a 79-year-old patient with prior C5-C6 ACDF who sustained a C6-C7 fracture with severe anterior displacement, treated with a posterior fusion at that level. These reports have all documented acute traumatic fractures to occur within two levels of a preexisting fusion, suggesting that areas immediately adjacent to a fusion are highly susceptible to instability.

Although acute traumatic fractures have been noted to occur in segments adjacent to a previous noninstrumented ACDF, the presentation of fractures directly through a fused construct has been largely unreported. Only one study in the literature has depicted this pattern of injury: Orndorff et al^9^ reported a single case of an acute C5-C6 facet fracture-dislocation after a motor vehicle accident in a 72-year-old man who underwent prior C3-C6 noninstrumented ACDF. As with our patient, treatment required a long C3-T2 instrumented posterior fusion. Yet, all reports in the literature of a prior noninstrumented ACDF with subsequent displaced fracture—either through the fusion mass or immediately adjacent to it—describe instability that occurred acutely after a traumatic event. We were unable to find any published cases reporting a subacute destabilization of the cervical spine from trauma in the setting of a previously solid noninstrumented anterior cervical fusion, highlighting the rarity of our patient's presentation. It is possible that the fracture pattern initially seen in our patient gradually displaced over time due to the altered anatomy in the cervical spine predisposed by a prior fusion. It is also possible that the displacement was overlooked by the initial diagnostic workup. Although the incidence of unrecognized spinal stability in trauma patients is quite low—according to a multi-institutional retrospective review, 0.21% in patients with a known spine fracture and less than 0.003% in all patients with trauma ^17^—it is quite plausible that a delay in diagnosis may be more likely in patients like ours. Our patient's initial CT scan was read as no fracture by the radiologist, and therefore, the patient was not evaluated by a spine surgeon while in the emergency department. Because of persistent neck pain, the patient was evaluated 10 days later by the senior author (C.W.C.). The consequences of a missed diagnosis may produce devastating results, from permanent neurologic impairment to even death. For this reason, it is crucial to analyze the diagnostic tools used to determine instability so that proper follow-up can be maintained in high-risk patients such as those with a prior cervical fusion.

Suspected instability of the spine should be investigated with a combination of a thorough clinical examination and radiographic findings. Symptomatic patients may report cervical pain, midline tenderness, and neurologic motor and sensory deficits. In the acute setting, emergency CT is the leading imaging modality of choice. Upright plain radiographs, although largely falling out of favor due to the advancements in CT, remain an important tool in visualizing cervical alignment and recognizing any vertebral displacements in the upright position. By illustrating the spine's load management and response to gravity in various dynamic states, standing, flexion-extension, and weight-bearing radiographs can be extremely useful in uncovering pathologies missed by CT, where the patient is supine. In patients with negative or ambiguous CT findings, Vincent and Anderson^18^ in a classic article recommend that the patient should be placed in a cervical collar with a follow-up examination that includes upright orthogonal or flexion-extension radiographs performed 2 weeks after the injury. It was observed that in neurologically intact patients with thoracolumbar fractures, plain radiographs changed eventual management from nonsurgical to surgical 25% of the time.^19^ A similar corollary may apply in the management of cervical spine fractures. In addition to their crucial role in revealing instability in the subacute outpatient setting, we believe that it may even be useful to obtain upright radiographs in the acute traumatic setting, if the initial CT scan was inconclusive. This may be especially applicable in patients with rigid fusion constructs—whether it be AS, DISH, or a previous noninstrumented anterior cervical fusion—whose altered spinal anatomy may predispose them to subtle injury. Using upright radiographs both in the initial assessment and in close follow-up may enable early recognition and timely treatment of injuries associated with increased mortality.

MRI may also serve as a useful adjunct in identifying instability, particularly when there is potential for ligamentous injury or infectious and neoplastic processes. The role of MRI in the context of the acute trauma may be underutilized, as CT of the cervical spine is often times the first and only image modality obtained. Although the increased sensitivity of cervical spine MRI for detection of soft-tissue injury and spinal cord injury without radiographic abnormalities is widely accepted, the value of MRI in the setting of a negative cervical spine CT remains a point of controversy. We have described the sequela of events that may occur in the setting of questionable CT findings in a patient who was ultimately found to have a cervical fracture through an noninstrumented fused segment with corresponding adjacent spinal cord stenosis. This begs the argument that MRI of the cervical spine, in the setting of a negative or questionable CT imaging, is beneficial in detecting missed cervical cord injuries that may have serious consequences in patient care. No consensus exists on the appropriateness of acquiring cervical spine MRI after CT imaging in the workup of acute cervical spine trauma; however, Onoue et al^20^ showed the significance in obtaining such imaging. The authors reviewed 7,301 patients who were admitted for blunt cervical spine trauma; CT imaging was obtained for all patients, however, MRI of the cervical spine detected significant injuries in 31% of patients who otherwise had a negative CT. In light of our experience as it relates to this specific case and suggestions found in the literature, it is reasonable to accept a greater role for routine MRI evaluation of the cervical spine, particularly if there is substantial clinical concern suggestive of occult injury.

## Conclusion

Multilevel noninstrumented fused cervical segments are susceptible to traumatic injury. Most cases in current literature report cervical spine fractures causing instability only at segments above or below the fusion rather than through it. Moreover, no cases in current literature describe a traumatic fracture in previously fused spines that gradually displaced over time. We report on a unilateral small, nondisplaced facet fracture sustained through a previously robust noninstrumented anterior cervical fusion that was complicated by posterior distraction with focal kyphosis causing cervical instability. Patients with preexisting noninstrumented fusions have a greater predisposition for atypical fracture patterns. We emphasize the importance of certain management approaches, including early upright radiographs as appropriate, that may prevent delays in diagnosis and minimize potentially devastating complications.
